# Effects of Carbon Nanotube Environmental Dispersion on an Aquatic Invertebrate, *Hirudo medicinalis*

**DOI:** 10.1371/journal.pone.0144361

**Published:** 2015-12-04

**Authors:** Rossana Girardello, Stefano Tasselli, Nicolò Baranzini, Roberto Valvassori, Magda de Eguileor, Annalisa Grimaldi

**Affiliations:** Department of Biotechnology and Life Sciences, University of Insubria, Varese, Italy; Jinling Institute of Technology, CHINA

## Abstract

The recent widespread applications of nanomaterials, because of their properties, opens new scenarios that affect their dispersal in the environment. In particular multiwall carbon nanotubes (MWCNTs), despite their qualities, seem to be harmful for animals and humans. To evaluate possible toxic effects caused by carbon nanotube environmental dispersion, with regard to aquatic compartment, we proposed as experimental model a freshwater invertebrate: *Hirudo medicinalis*. In the present study we analyse acute and chronic immune responses over a short (1, 3, 6 and 12 hours) and long time (from 1 to 5 weeks) exposure to MWCNTs by optical, electron and immunohistochemical approaches. In the exposed leeches angiogenesis and fibroplasia accompanied by massive cellular migration occur. Immunocytochemical characterization using specific markers shows that in these inflammatory processes the monocyte-macrophages (CD45^+^, CD68^+^) are the most involved cells. These immunocompetent cells are characterized by sequence of events starting from the expression of pro-inflammatory cytokines (in particular IL-18), and amyloidogenensis. Our combined experimental approaches, basing on high sensitive inflammatory response can highlight adverse effects of nanomaterials on aquatic organisms and could be useful to assess the MWCNTs impact on aquatic, terrestrial animal and human health.

## Introduction

The use and manufacturing of nanomaterials are paralleled with a rapid expanding of their environmental discharge that are expected to be important in determining toxicity. These nanoscale pollutants are non-biodegradable and for this reason, it is difficult to clean up once the environment is contaminated. Nanoparticles (NPs) can be found mixed in the air, in the soil and more often being washed from the soil into the water (rivers and lakes) being harmful to the health of many animals, including humans [1,2].

Although some researches have shown a lack of toxic effects [3], several studies have reported potential impacts of carbon nanotubes (CNTs) on both aquatic and soil organisms which can uptake nanomaterials via skin contact or oral uptake through the gastrointestinal tract [4,5]. CNTs dissolved in water and accumulate in soils through accidental spills, application of sewage sludge, deposition of airborne manufactured nanoparticles, use of manufactured nanoparticles in agrochemicals or soil remediation. [6], can induce toxic effects in different organisms [7–10] or can be accumulated in several animals such as earthworms [11–13], gastropod molluscs [4], zooplankton [14–17]. However, for these nanomaterials the mechanisms determining the toxicity are still unclear. CNTs toxicity deriving from mammalian tests show that they are cytotoxic and genotoxic for different types of cells such as macrophages where the exposure induces the release of reactive oxygen species (ROS) [18], necrosis, chromosomal aberrations, apoptosis [19] and inflammatory cytokine expression, such as IL-8 [20]. Therefore studying the NPs evoked inflammatory processes could be crucial to understand the potential effects of nanomaterial as stressor on organisms.

In this work, we evaluate the inflammatory response induced in the leech *Hirudo medicinalis* after in vivo exposure to MWCNTs. The interest in using this animal model is due to its anatomical and physiological features that allows to observe and study events linked to the cellular immune response. In particular, it can be easily and unambiguously evaluated in leech’s body wall, which is a predominantly avascular muscular district containing a few immunocompetent cells of myeloid origin, i.e. macrophages, granulocytes and NK [21]. In addition our previous papers [22–27] and a recent report [28] indicated the existence in leech of several CDs (cluster of differentiation) proteins, similar to mammalian CDs, which can be used as markers to easily identify cells involved in the immune response. Once the leech innate immune systems recognizes foreign antigens, the responses against non-self material, lesion or bacterial challenge in the body wall are rapidly induced (24 hours) and can be studied by morphological and histochemical analyses [21,26].

Our data indicate that in leech MWCNTs, induce macrophage recruitment and amyloid deposition, highlighting the potential risks for public health link to carbon nanotubes aquatic environmental diffusion.

## Materials and Methods

All experiments were performed in triplicate.

### MWCNTs preparation

Commercially available and industrially employed Nanocyl Thin Multi-wall Carbon Nanotubes NANOCYL^™^ NC7000 were obtained from NANOCYL (Belgium, Sambreville). The MWCNTs have an average 9.5 nm external diameter by 1.5 μm mean length with surface area of 250–300 m^2^/g. They were manufactured by a CCVD (catalytic carbon vapor deposition) process with a purity of 90% C and 10% metal oxide, of which 9.6% was aluminum oxide with traces of iron and cobalt [29]. In these experiments, the pristine MWCNTs were used directly without any chemical processing before use. MWCNTs powder was weighed, dissolved in water and sonicated in an ultrasonic bath 15 min for two cycles to avoid aggregation of particles. The concentration MWCNTs was determined basing on previous data in literature [30] reporting specific biochemical parameters alteration (i.e. mitochondrial enzymatic activity) after in vivo MWCNTS exposure.

### In vivo study design

Leeches (*H*. *medicinalis*, Annelida, Hirudinea, from Ricarimpex, Eysines, France), measuring 10 cm, kept in water at 20°C in aerated tanks and fed weekly with calf blood. *H*. *medicinalis* were exposed to [400mg/L] MWCNTs powder dissolved in water. Animals extensively agitate the water as part of their normal activities, generating a continuous re-suspension of nanotubes, preventing their aggregation in water, and thereby continuously exposing their body wall. The model reflects that associated to aquatic animals that can be subjected to an uncontrolled direct and indirect exposition to CNTs. Animals were randomly divided into separate experimental groups (five individuals for each time points) and exposed to MWCNTs for 1, 3, 6 e 12 hours, to evaluate the acute response to treatment, and for 1, 2, 3, 4 and 5 weeks to value chronic response. Controls consisted of animals that were kept similarly without MWCNTs in the water. Before sacrifice, control and MWCNTs exposed at specific time points leeches were anesthetized with an 10% ethanol solution and then dissected to remove body wall tissues at the level of 20^th^ metamere.

### Assessment of internalization of MWCNTs and metal oxide in leech tissues

Internalization of MWCNTs in leeches was assessed by the following procedure: portions of tissue samples obtained after MWCNTs environmental exposure were excised and digested in 5 N potassium hydroxide (KOH) over night at room temperature, then washed repeatedly in distilled water (dH_2_0) to remove potassium salt, resuspended in 100 μl of dH_2_O and dried on a copper grids, (Formvar Carbon Film) for transmission electron (TEM) analysis (Jeol JEM 1010, Tokyo, Japan). Images were acquired with Morada, Olympus (Tokyo, Japan) digital camera. To show that the KOH treatment does not affect MWCNTs structure, crude powder was treated in the same way of tissue sample. As control, pristine MWCNTs were re-suspended in dH_2_O and observed.

### Optical and Trasmission Electron Microscopy (TEM)

Tissues from leech body wall, were fixed for 2 h in 0.1 M cacodylate buffer at pH 7.4, containing 2% glutaraldehyde. Specimens were then washed in the same buffer and post-fixed for 1 h with 1% osmium tetroxide in cacodylate buffer, pH 7.4. After standard ethanol dehydration, specimens were embedded in an Epon-Araldite 812 mixture. Sections were obtained with a Reichert Ultracut S ultratome (Leica, Wien, Austria). Semi-thin sections (0.75 μm in thickness) were stained by conventional methods (crystal violet and basic fuchsin, according to Moore et al. [31]) and subsequently observed under the light microscope Nikon Eclipse Ni (Nikon, Tokyo, Japan). Data were recorded with a DS-5M-L1 digital camera system Nikon. Ultrathin sections (80 nm in thickness), stained by uranyl acetate and lead citrate, were observed with a Jeol 1010 EX electron microscope. Data were recorded with a MORADA digital camera system (Olympus).

### Scanning electron microscopy (SEM) and X-ray spectroscopy (EDS)

To obtain three-dimensional imaging by SEM, tissues from leeches untreated or exposed to MWCNTs were fixed in 4% paraformaldehyde for 1 h at room temperature. The specimens, washed in PBS (pH 7.2), were dehydrated in an increasing series of ethanol, cleared in in xylene for 30 minutes and then penetrated with paraffin (melting point, 58–60°C, Bioptica, Milan, Italy), at 60°C over night. Paraffin sections (7 μm) were deparaffinized with xylene and dehydrated in an increasing series of ethanol. Slides were mounted on stubs, sputter coated with a thin layer of gold and then observed with a SEM-FEG XL-30 microscope (Philips, Eindhoven, The Netherlands).

To confirm the presence of aluminum, iron and cobalt associated to the crude MWCNTs powder and evaluate their entrance in leech tissues, samples were observed in backscattered electron mode with a scanning electron microscope coupled with an energy dispersive X-ray analyzer (EDAX Genesis 2000). Samples were stuck onto slide holders and sputter coated with a thin layer of gold. Photographic maps of element distribution obtained on the image frames were processed by Image Analysis (1994) (Soft-Image Software GmbH). These maps were then superimposed to each source image with Adobe Photoshop (Adobe Systems).

### Atomic Absorption Spectroscopy (AAS)

Chemicals used for the preparation of all standard and sample solutions were metal trace analysis grade: MilliQ water (Millipore) and HCl (Baker 9530, 36.5–38%). The calibration standard solutions were prepared from a 1000 mg/L standard solutions (J.T. Baker Instra-Analyzed), and the blanks were prepared with 0.1M HCl.

All measurements were performed on a Solaar M6 atomic absorption spectrometer (Thermo Fisher): Al, Fe and Co were determined with a graphite furnace (GFAA) coupled with Zeeman background correction. Wavelength, bandpass and all others instrumental parameters were set according manufacturers recommendations.

The conventional instrumental detection limits (IDL, based on three standard deviations of the Blank signal) were calculated for each analytical run, typically ranging 1–3 ng/L.

The reported results are the mean of three measurements.

### Immunohistrochemistry, enzimatic hystochemistry and Thioflavin-T staining

Leech tissues were embedded in polyfreeze tissue freezing medium (OCT, Tebu-Bio, Italy) and immediately frozen in liquid nitrogen. Cryosections (7 μm in thickness) were obtained with a Leica CM 1850 cryotome and slides were immediately used or stored at -20°C.

For indirect immunofluorescence, cryosections were rehydrated with phosphate buffer saline (PBS) for 5 min at room temperature and then incubated for 30 min in a blocking solution with 2% BSA (Bovine Serum Albumin) and 0.1% Tween20 in PBS. Subsequently, sections were incubated for 1h at room temperature with the following polyclonal primary antibodies diluted 1:200 in blocking solution: rabbit anti-human CD45 (Twin Helix, Milano, Italy), which reacts, as previously demonstrated, with leech hematopoietic precursors cells and myeloid leukocytes cells [24]; rabbit anti-human CD68 (Santa Cruz Biotechnology, CA, USA) which reacts, as previously demonstrated, with leech macrophage-like cells [26,32] and rabbit anti-human IL-18 (Abnova, Germany). After washing, sections were incubated for 1h at room temperature with the secondary antibody donkey anti-rabbit Cy3-conjugated (excitation 562 nm, emission 576 nm), diluted 1:200 (Jackson ImmunoResearch Laboratories, Inc., West Grove, USA). After further washing with PBS, cryosections were incubated for 15 min with the nuclear marker DAPI (4',6-diamidino-2-phenylindole). Then, slides were mounted with Cytifluor (Cytifluor, USA). In control experiments, primary antibodies were omitted and sections were treated with BSA-containing PBS and incubated only with the secondary antibodies.

For acid phosphatase (ACP) detection, cryosections were rehydrated with PBS for 5 min, incubated with 0.1 M sodium acetate-acetic acid buffer for 5 min and then treated for 1 hour and 30 min at 37°C with the reaction mixture (0.1 M sodium acetate-acetic acid buffer, 0.01% naphtol AS-BI phosphate, 2% NN-dimethylformamide, 0.06% Fast Red Violet LB and 0.5nM MnCl2). After washings in PBS, the slides were mounted with PBS/glycerol 2:1.

According to Grimaldi et al. [33], amyloid fibrils were specifically highlighted using fluorescent dye Thioflavine T (excitation wavelength of 465 nm emission) [34]. For double staining Thioflavin-T/CD-68, the Thioflavin-T method was first applied, followed by the immunedetection of anti-CD68 antibody.

Samples were examined by light/fluorescence microscope Nikon Eclipse Ni (Nikon, Japan) and pictures were collected with the digital camera Nikon D5-5M (Nikon). Images were combined with Adobe Photoshop (Adobe Systems, Inc.).

### Statistical Analysis

The number of migrating ACP^+^ cells were counted in each section of three independent experiments for each time lapse using the Image J software package (http://rsbweb.nih.gov/ij/download.html). The number of counted cells is standardized on the untreated leeches. Statistical significance was assessed by an unpaired Student’s t test using Origin 5.0 software (Microcal).

### Protein extracts preparation, SDS-PAGE and Western Blot

*H*. *medicinalis* tissues from the body wall were frozen in liquid nitrogen and then homogenized with a mortar. For SDS-PAGE, leech homogenates were suspended in extraction buffer (2X Laemmli's Buffer in the presence of a protease inhibitor cocktail (Sigma, Milan, Italy). The particulate material was removed by centrifugation at 13000 rpm for 10 min at 4°C in a refrigerated Eppendorf Minispin microcentrifuge. Supernatants were denatured at 100°C for 10 min.

Equal amounts of protein extracts were separated in analytical SDS-PAGE using 10% acrylamide minigels. Molecular weights were determined by concurrently running broad range standards from Bio-Rad (Bio-Rad, Richmond, MA, USA). Proteins separated by SDS-PAGE were transferred onto Bio-Rad nitrocellulose filters. Membranes were then saturated with 5% non fat dried milk in Tris buffered saline (TBS, 20 mM Tris-HCl buffer, 500 mM NaCl, pH 7.5) at room temperature for 2 hr, and incubated for 90 min with rabbit polyclonal anti-CD45 antibody (1:500 dilution in 5% TBS-milk). After washing the membrane three times with TBS-Tween 0,1%, the antigens were revealed with the secondary anti-rabbit IgG antibody HRP-conjugated (Jackson ImmunoResearch Laboratories), diluted 1:5000. After washing, the immunocomplexes were revealed with luminol LiteAblot^®^ PLUS Enhanced Chemiluminescent Substrate (EuroClone S.p.A., Pero, Italy). In control experiments, anti-CD45 antibody was omitted. Bands were normalized, using the ImageJ software package (http://rsbweb.nih.gov/ij/download.html), with the housekeeping protein GAPDH, which was detected with a rabbit polyclonal anti-human GAPDH IgG (Proteintech^™^, Chicago, USA) diluted 1:2000. The expression level of CD45 in exposed leeches was reported relatively to control untreated animals. Experiments were performed in triplicate and data represent the mean values ± SEM. Statistical significance was assessed by an unpaired Student’s t test.

## Results

### Determination of MWCNT presence in tissues

**Fig 1:**
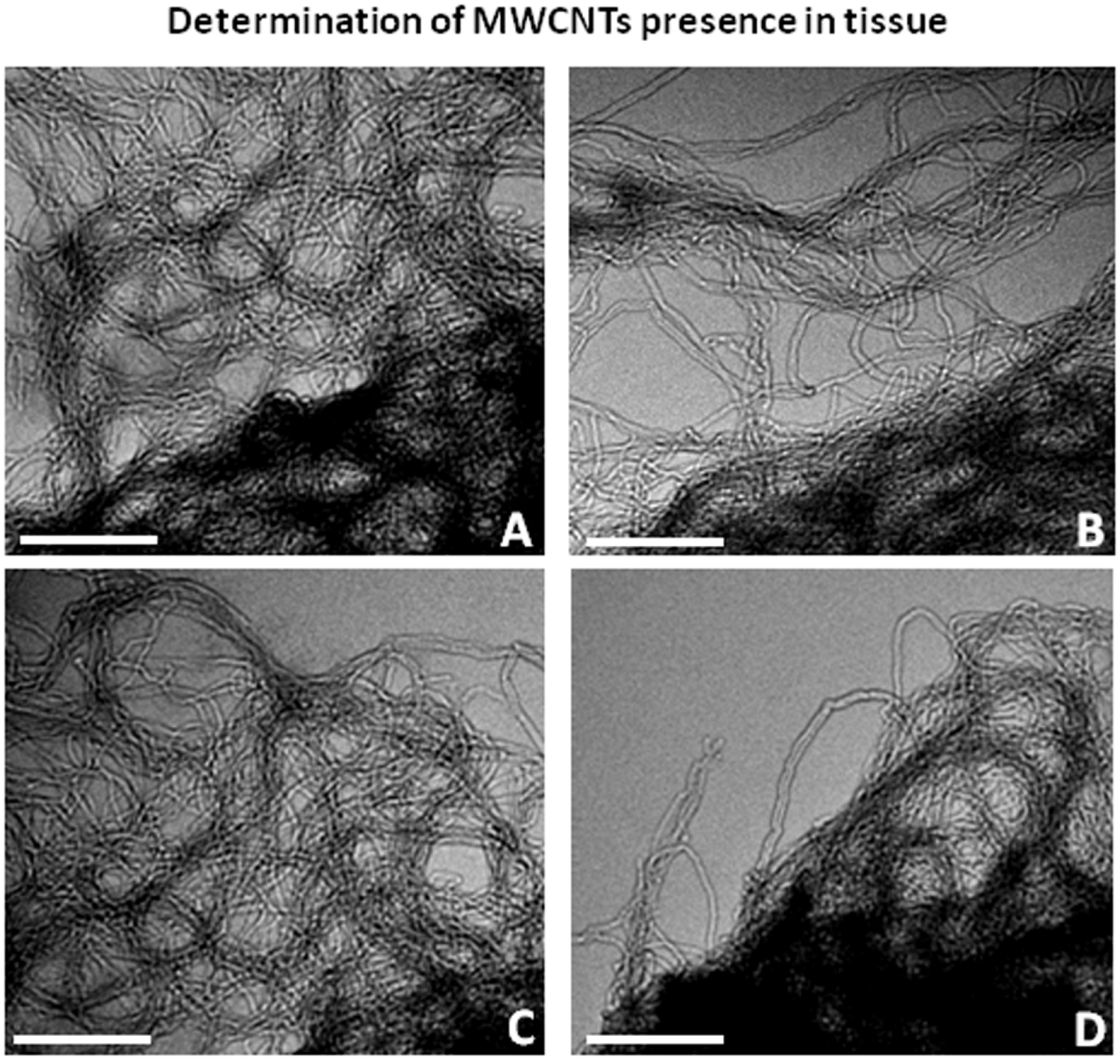
Determination of MWCNTs presence in tissues (A-D). (A) MWCNTs extracted by means of KOH digestion from exposed-animals tissues. (B-D) MWCNTs crude powder (used as control) raw (B), after sonication (C) and after KOH treatment (D). Bars in A-D: 500nm.

The internalization of MWCNTs has been first validated by means of KOH digestion tissues derived from exposed animal (for detail see experimental procedure in the material and method section). TEM analysis showed that nanotubes from digested exposed-animals tissues (Fig 1A) had the same characteristics of MWCNT crude powder, used as control Fig 1B). Furthermore, sonication and KOH treatment do not affected MWCNTs morphology (Fig 1C and 1D).

### Morphological analysis of untreated and MWCNT exposed leech tissues

**Fig 2:**
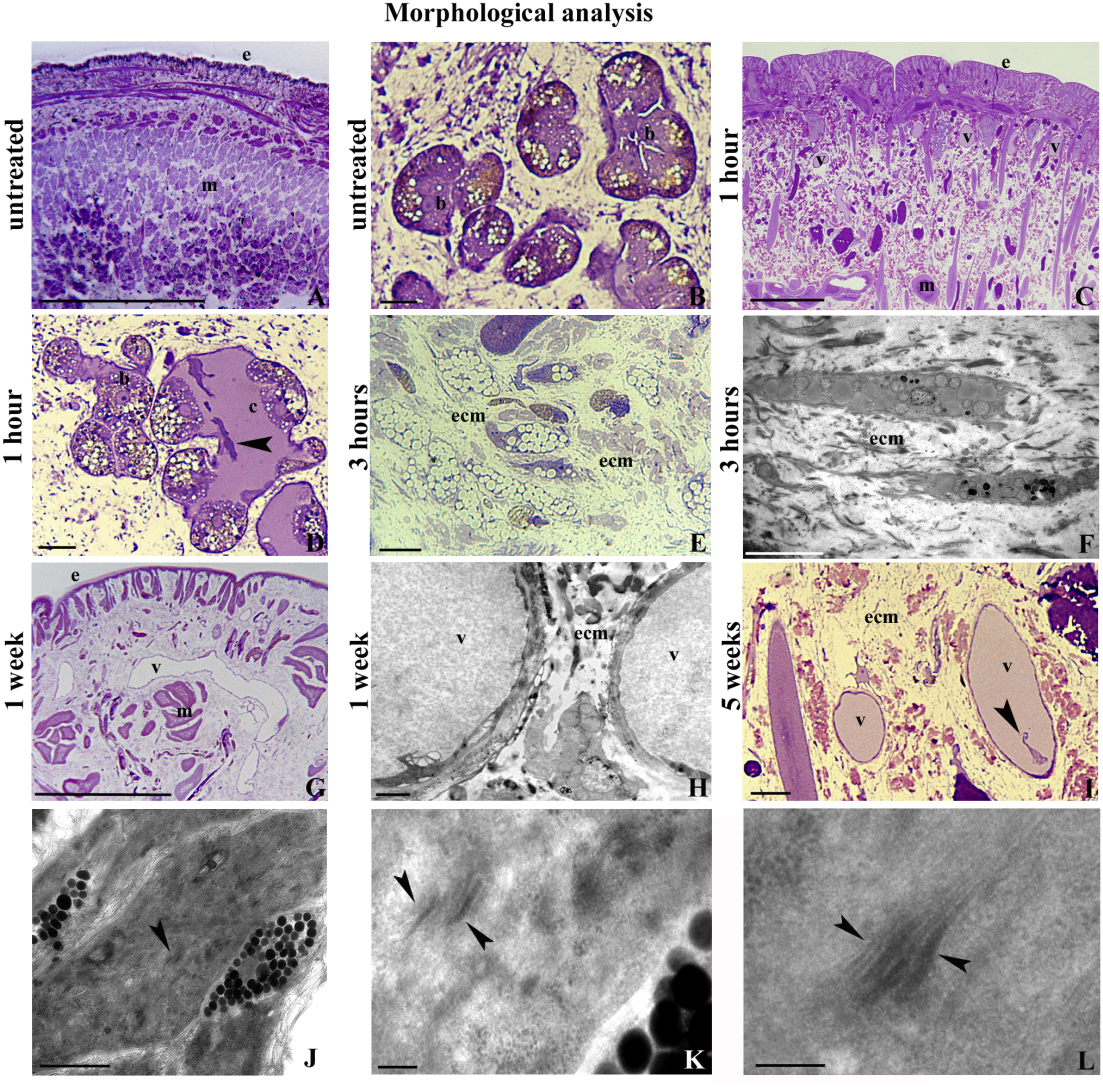
Morphological analysis (A-L). Morphological at optical (A-E, C, I) and transmission electron microscopes (F, H, J-L) analyses of sections from *H*. *medicinalis* body wall. The body wall of untreated leeches results practically avascular (A) and the botryoidal tissue (b) appears as a solid chord of clustered cells (B). After 1 hour from MWCNTs treatment, numerous neo-vessels (v) are found among muscles (m) and under the epithelium (e) (C). Within the new cavities (c) lined by the botryoidal tissue (b), immunocompetent circulating precursors cells (arrowhead) are clearly distinguishable (D). After 3 hours from treatment, numerous fibroblasts are visible immersed in an abundant extracellular matrix ECM (E, F). After 1 (G, H) up to 5 weeks (I) from MWCNTs treatment, numerous vessels (v) and fibroblasts (arrowheads) are still visible in the body wall. (J-L) Detail of MWCNTs (arrowheads) freely dispersed in the cytoplasm of macrophage-like cells. Bars in A, C, G: 100μm; Bars in B, D-E, I: 10μm; Bar in F: 5μm; Bar in H: 2μm; Bar in J: 500nm; Bars in K-L: 200nm.

*H*. *medicinalis* body wall is made of an epithelium enwrapping thick layers of muscle fibers packed in groups (Fig 2A). Under the muscular sac, virtually avascular, there is the botryoidal tissue embedded in the parenchyma localized between the gut and the body wall (Fig 2A). Starting from 1h up to 12 h after MWNTs treatment, a network of blood vessels were evident in the thickness of muscle-cutaneous sac (Fig 2C). The angiogenic process, typical of inflammatory phase, is considered part of innate immune responses. The formation of new vessels is due to a remodeling of the botryoidal tissue in which, by a dehiscence process, a lumen and immunocompetent circulating cells became visible (Fig 2D). Parallel a massive fibroplasia affecting the entire body wall was observed. Leech fibroblasts, responsible for synthesis and remodeling of the extracellular matrix, were spindle-shaped, with numerous lipid droplets in the cytoplasm and laminar projections forming a microenvironment where fibrillogenesis occurred (Fig 2E and 2F).

Analyses at optical microscope showed that the inflammatory state, already observed in short-time-treated animals, also persisted after a prolonged period of MWCNTs exposure. Starting from 1 up to 5 weeks of MWCNTs treatment, the muscle-cutaneous sac were highly vascularized (Fig 2G–2I) and infiltrated by a large number of cells (Fig 2G, 2H and 2I) derived from circulating cells as previously demonstrated [24]. Ultrastructural analysis at TEM showed that particulate acquisition was evident as engulfment of particles settled in the cytoplasm of infiltrating cells showing typical macrophage features (Fig 2J–2L), as we recently demonstrated [35]. These data suggest that macrophages constitute the cells primarily involved in the recognition of this exogenous (non self) material.

### Characterization of cells involved in the inflammatory response caused by MWCNTs exposure

**Fig 3:**
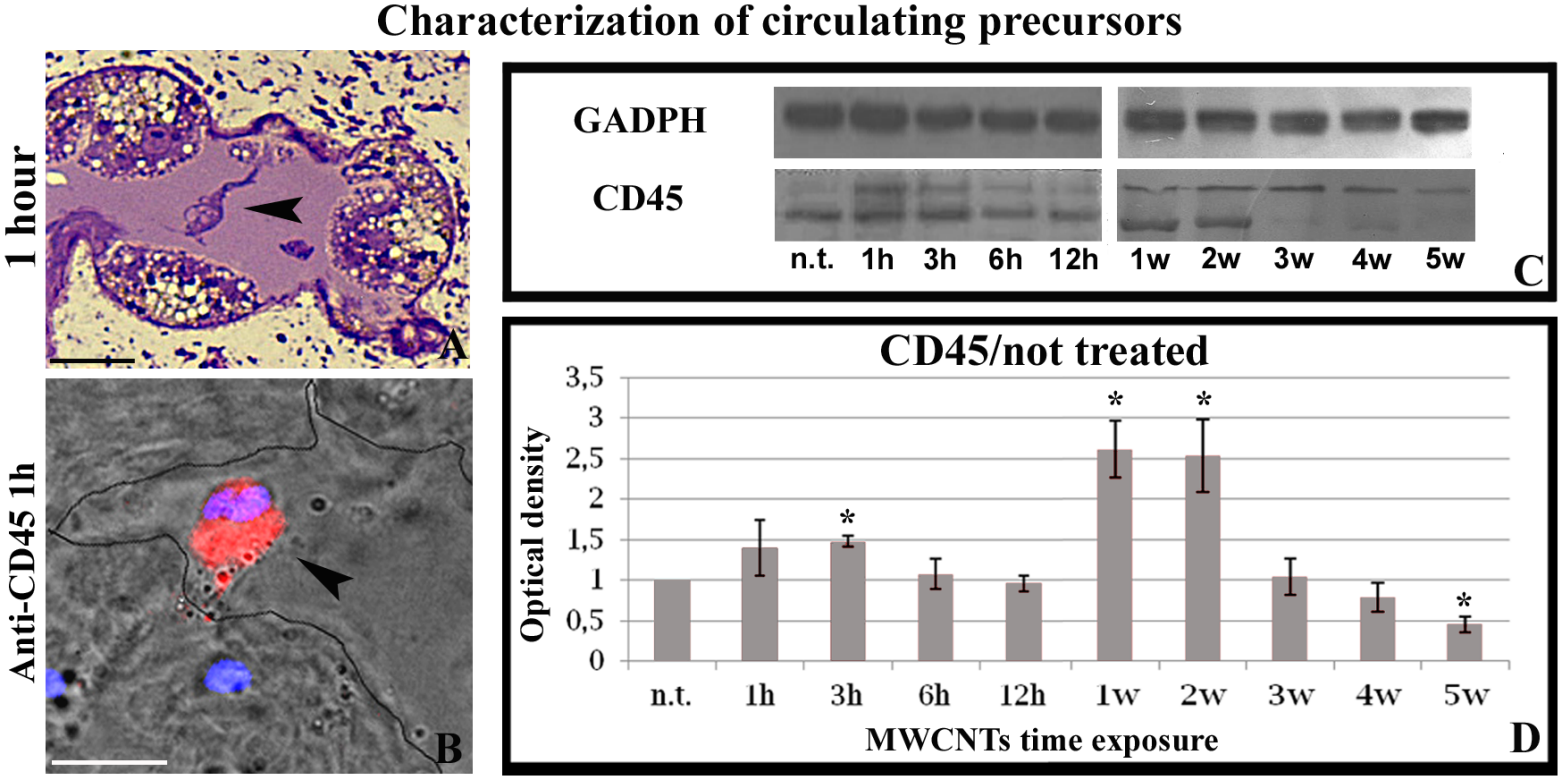
Characterization of circulating precursors cells (A-D). Morphological (A) and immunohistochemical (B) analysis of cryosections from *H*. *medicinalis* body wall. The circulating precursors (arrowhead) visible in lumen of neo-vessels are highly positive for anti-CD45 antibody. Nuclei were counterstained with 4,6-diamidino-2-phenylindole (DAPI; blue). (C-D) Western blot analysis. A protein extracts from the body wall of untreated (n.t) and MWCNTs treated leeches from 1 to 12 hours and from 1 to 5 weeks were probed with the anti-CD45 antibody. The housekeeping protein D-glyceraldehyde-3-phosphate dehydrogenase (GAPDH) was used as a loading control. In all samples, the anti-CD45 detected specific immunoreactive band 24 of about 145 kDa (C), according to the molecular weight ladder (kDa). CD45 protein was quantified by densitometry from three experiments. *P<0.05 compared with untreated leeches (D). Bars in A-B: 10μm.

In order to characterize the cell types recruited in the muscle body wall after MWCNTs exposure and to confirm the hypothesis that MWCNTs can induce an immune response activation, immuno-staining experiments, enzymatic histochemistry and colorimetric analyses were performed on cryosections of tissues collected starting from 1 hour up to 5 week after treatment. Immunofluorescence experiments confirmed that the circulating cells, within the neo formed lumen (Fig 3A) and in the peripheral vessels (Fig 3B), were positive for the antibody anti-CD45, the marker commonly used to identify vertebrates leukocytes and leech precursors of circulating cells [24]. The expression of CD45 in control and in MWCNTs treated animals was confirmed by Western blot assay (Fig 3C). Immunoblot analysis of leech body wall extracts validated the presence of two immunoreactive products of about 145 kDa and 180 kDa. These molecular weights were consistent with those found in vertebrates, in which different isoforms of CD45 has been identified with a molecular weight ranging from 140 kDa to 240 kDa [36]. The expression profile of the two isoforms varied in relation to the timing of nanotube administration (Fig 3D). 1 hour after MWCNT treatment, the 180 kDa isoform expression increased significantly in respect to the basal expression level of untreated leeches while the expression of the 145 kDa isoform dramatically decreased starting from 3 week after MWCNTs exposure. Summarizing, the total amount of CD45 expression showed a cyclical pattern, with a peak of expression after 3 hours and after 1 week from treatment. GAPDH was used as internal reference and bands intensity appeared homogeneously distributed in the loaded samples. No specific signals were observed on the negative control experiments performed omitting the primary antibody.

**Fig 4:**
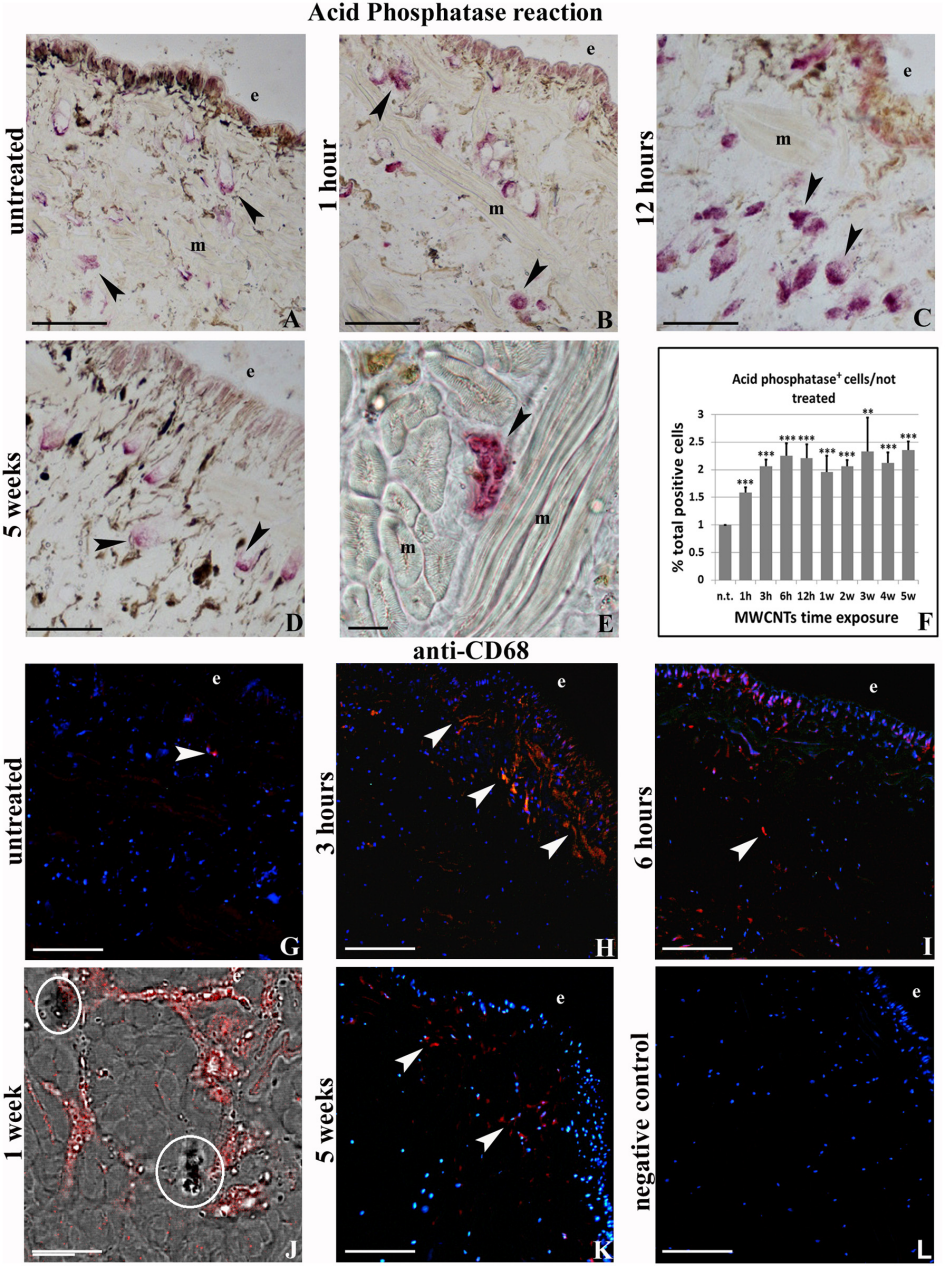
Acid phosphatase reaction (A-F) and anti CD68 immunofluorescence staining (G-L). Resident in untreated (A, G) and migrating macrophages-like cells in treated leeches (B-E) and (H-L), located under the epithelium (e) and among the muscle fibers (m), are positive for acid phosphatase reaction (arrowheads in A-F) and for anti-CD68 (G-L). (F) Quantitative evaluation of cell numbers. Column 1: number of cells in untreated sample, columns 2–10: number of cells in MWCNTs treated sample from 1h up to 5 weeks. *p<0.01. (J) Combined transmission and fluorescence images showing CD68^+^ cells (in red) in spatial association with MWCNTs aggregates (circled). Bars in A-E, G-I, K-L: 100μm; Bar in F, J: 10μm.

**Fig 5:**
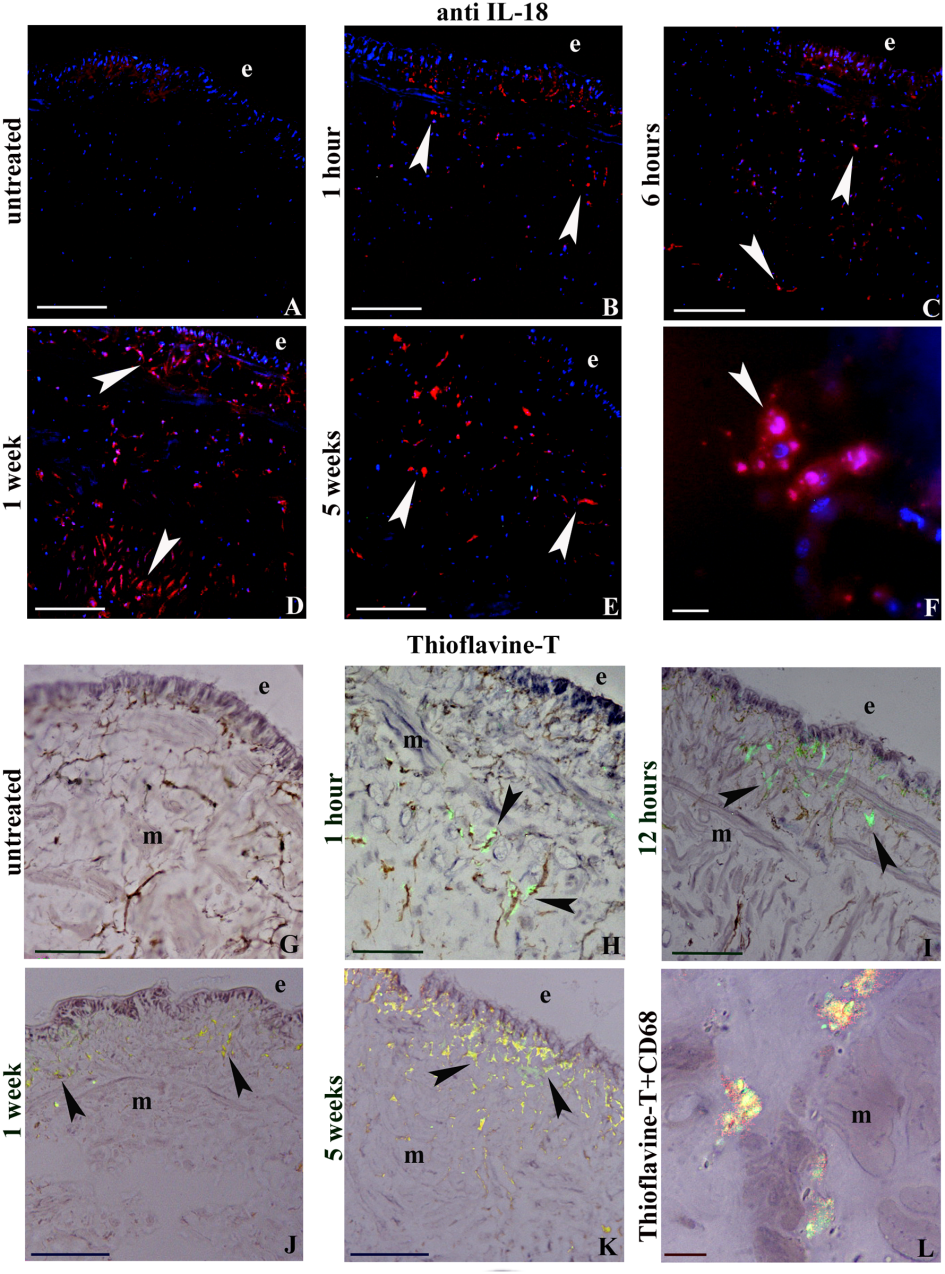
Anti-IL18 (A-F) and Thioflavine-T staining (G-L). (A-F) Localization of IL-18. Note the population of resident in untreated (A) and migrating (B- F) immune-responsive cells (arrowheads) located under the epithelium (e) and among the muscle (m). Nuclei are counterstained with DAPI (blue). (G-K) Thioflavin-T method. Amyloid material is stained in yellow (arrowheads). (L) Double-staining of Thioflavin-T (yellow) and macrophage markers CD68 (red) in a cryosection of 3 hours MWCNTs treated leech body wall. Bars in A-E, G-K: 100μm; EDS analysis.

To characterize the large amount of cells infiltrating the extracellular matrix among the muscle fibers and the new vessels, histo- and immuno-cytochemical enzymatic approaches were used. Unlike control leeches, in animals exposed to MWCNTs, starting from 1 hour up to 5 weeks, an increased amount of migrating cells were observed in the muscle body wall. Numerous migrating cells were positive for the acid phosphatase reaction (Fig 4A–4E), selectively staining lysosomal enzyme of cells with phagocytic activity, such as macrophages. Results showed that the number of migrating cells increased after 1h from MWCNTs exposure, the highest value was reached after 3h and persisted even after 5 weeks from treatment, as demonstrated by cell counting performed on 3 representative images of each time lapse, and was statistically significant (Fig 4F). The same cells, visible underneath the epithelium and migrating towards the MWCNTs aggregates located in the muscular layer, expressed the typical macrophage marker CD68 (Fig 4G–4L) and the evolutionarily highly conserved pro-inflammatory cytokine IL-18 [37,38] (Fig 5A–5F). We have previously demonstrated, both in vertebrates and invertebrates, the link among stress condition, immune responses and amyloid fibril production [33,39]. Here we verified whether MWCNTs were able to induce the production of amyloid fibrils as well. By using the specific Thioflavin-T colorimetric method (Fig 5G–5L) and performing double localization CD68/Thioflavin-T experiments, we confirmed macrophages as main producers of amyloid fibrils (Fig 5L).

### EDS and AAS analyses

**Fig 6:**
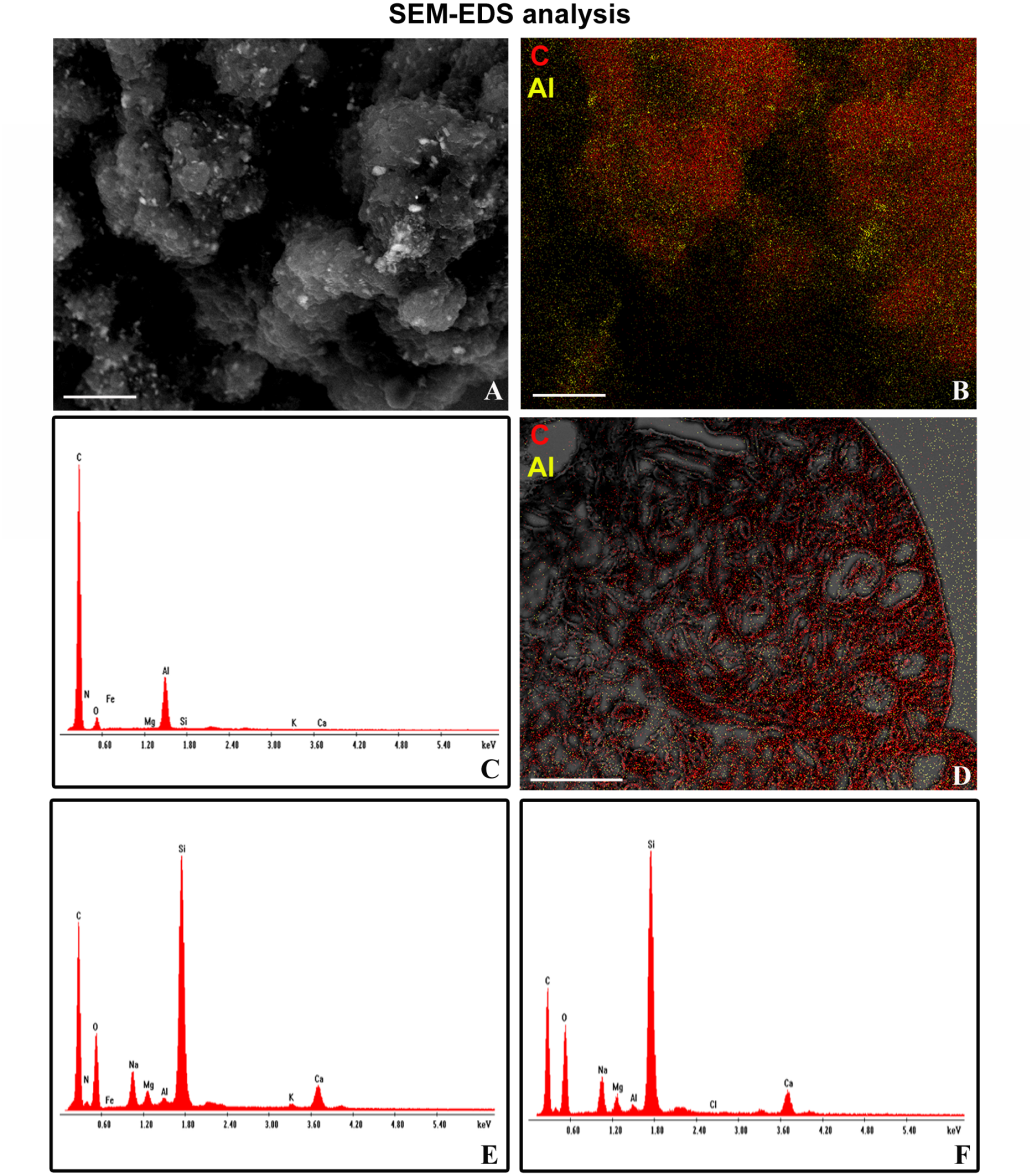
**SEM-EDAX analysis (A-F).** SEM (A) and Elemental mapping images (B) of MWCNTs crude powder (red: Carbonio; yellow: Aluminum). EDAX spectrum for MWCNTs crude powder (C). Combined SEM-EDAX elemental mapping image of a section of *H*. *medicinalis* body wall 5 weeks after treatment (D). EDAX spectra for treated (E) and untreated (F) tissue samples. Bars in A, B: 10μm; bar in D: 100μm.

EDS analysis was performed in order to confirm the possible presence, in the leech exposure solution, of metal oxide impurities associated to MWCNTs and to describe their possible entrance in leech tissues. Even if microanalytical EDS analysis confirmed the presence of aluminum associated to MWCNTs aggregates (Fig 6A–6C), no signal was detected for this metal in leech tissues after MWCNTs exposure (Fig 6D–6F). No picks were instead detected for iron and cobalt (Fig 6C, 6E and 6F).

The concentration of aluminum oxide in leech exposure water, assessed by AAS was, of 0,25 μg/L, while the concentration of iron and cobalt were below the IDL (instrumental detection limits).

## Discussion

Every exposure to different contaminants, such as organic compounds [30,40,41] or metals [42–45], increases the risk of damage to tissues, cells, DNA and other vital molecules. For the mentioned stressors each exposure potentially can cause programmed cell death, genetic mutations, cancers, immune and endocrine system disorders [46]. With respect to nanomaterials, after decades of research, much remains unknown about how MWCNTs interact with cell behaviors [47,48]. Our data suggest that more attention should be required controlling not only the use and manufacturing of engineered nanomaterials but also their environmental discharge. Indeed, utilizing an animal model with a relative simple anatomy, in this work we demonstrated that MWCNTs exposure can stimulate a response of the innate immune system of organisms. The evidenced strong inflammatory responses induced in leeches by nanotube exposure, suggests that this nanomaterial is able to penetrate superficial barriers, and to promote angiogenesis, fibroplasia, massive migration of immune cells, and pro-inflammatory cytokines such as IL-18 in turn linked to amyloid fibril formation.

Internalization of MWCNTs is validated by KOH digestion of tissues and the gross accumulation of MWCNTs in the leech tissues indicates that they mainly enter through the skin. Moreover, optical and ultrastructural analysis at TEM confirm the presence of MWCNTs aggregates in the muscular layer and dispersed in the cytoplasm of macrophage-like cells. After MWCNTs exposure, we observed a massive remodeling of extracellular matrix that plays a key role in terms of immune response since the collagen is reorganized to form a scaffold that drives and supports new vessels branching and immunocompetent cells migration [25,49].

AAS analysis confirmed that the concentration of aluminum oxide in the leech exposure solution is even lower than that accepted for human health in drinking water (as reported in the guide line of World Health Organization 2011 [50]) and no metals, such as aluminum, cobalt and iron, were detectable in leech tissues by EDS analysis. These data confirm that the tested responses in leeches are caused by MWCNTs and not by metal oxide impurities in the exposure solution. Our data are also supported by previous finding in rodents, showing that MWCNTs were capable of producing inflammation and fibrosis in different tissues regardless of the process by which they were synthesized and the types and amounts of metals they contained [1,51].

*H*. *medicinalis*, like other invertebrates, has an innate immune system which provides for a nonspecific response characterized by proliferation and migration of immune cells towards stimulated area. In leeches the innate immune system utilizes the cells of myeloid lineage such as macrophages, granulocytes and NK performing different types of responses in relation to the antigen. Small dimensions antigens, i.e. bacteria penetrating into the body wall, are phagocytized while larger ones induce both cytotoxic response and encapsulation. In particular, after injury or bacterial injection a massive migration of macrophages towards stimulated region occurs [26,52]. The same responses are observed in leeches after MWCNTs exposure. The undifferentiated precursors, expressing the common leukocyte marker CD45, are conveyed by vessels to the body wall where they can leave the bloodstream. Here they differentiate into mature CD68 positive cells with migratory and phagocytic capacity, as demonstrated by their positivity to the acid phosphatase reaction [24]. The different expression of CD45, at various time of exposure, leads to hypothesize that a cyclic call of monocytes on the inflammation site to maintain high and constant the macrophage number occurs. As we previously demonstrated [26,53], the cytokines released by macrophages, endothelial cells and fibroblasts, are potent angiogenic factors which stimulate chemo-attraction, proliferation, secretion and cell migration. Recently a new player in innate immune responses has been identified and amyloidogenesis is proposed as a fundamental detoxifying event [39] that during evolution also acquired physiological additional functions packaging melanin and driving the pigment towards a non-self-invader both in vertebrates [54] and in invertebrates [33,55].

Here we find that, after MWCNTs exposure, leech CD68 positive macrophages produce amyloid material, as demonstrated by Thioflavin-T staining. Recently, a correlation between amyloidogenesis and the production of IL-18 (a cytokine highly conserved and responsible for activation and regulation of innate immune system) has been demonstrated [37,38]. We observed that in leeches the expression of IL-18 in concomitance with Thioflavin-T signals increases in a time-dependent way and progressively with MWCNTs exposure time. Here we suggest that in leeches IL-18 and amyloid material production are associated processes within macrophage cell activation.

## Conclusions

Our combined experimental approaches, basing on high sensitive inflammatory response can highlight adverse effects of nanomaterials on aquatic organisms. These simple experimental approaches and the use of an anatomically simple model such as leeches, where the effects of stressful events are unequivocally interpretable, are an important tool that can be finalized to monitor the diffusion of MWCNTs in the water environment and the effects of this nanomaterial as stressor on organisms.
